# Cytotoxic Activity and Docking Studies of 2-arenoxybenzaldehyde N-acyl Hydrazone and 1,3,4-Oxadiazole Derivatives against Various Cancer Cell Lines

**DOI:** 10.3390/molecules27217309

**Published:** 2022-10-27

**Authors:** Esranur Aydın, Ahmet Mesut Şentürk, Hatice Başpınar Küçük, Mustafa Güzel

**Affiliations:** 1 Center of Drug Discovery and Development, Research Institute for Health Sciences and Technologies SABITA, Istanbul Medipol University, 34810 Istanbul, Turkey; 2 Department of Molecular Medicine, and Biotechnology, Health Sciences Institute, Istanbul Medipol University, 34810 Istanbul, Turkey; 3 Faculty of Pharmacy, Department of Pharmaceutical Chemistry, Istanbul Biruni University, 34010 Istanbul, Turkey; 4 Department of Chemistry, Faculty of Engineering, Organic Chemistry Division, Istanbul University-Cerrahpasa, 34320 Istanbul, Turkey; 5 Department of Basic Pharmaceutical Sciences, School of Pharmacy, Istanbul Medipol University, 34810 Istanbul, Turkey

**Keywords:** hydrazone derivatives, oxadiazole derivatives, A-549, MDA-MB-231, PC-3, anticancer activity, docking, molecular modeling studies

## Abstract

To understand whether previously synthesized novel hydrazone and oxadiazole derivatives have promising anticancer effects, docking studies and in vitro toxicity assays were performed on A-549, MDA-MB-231, and PC-3 cell lines. The antiproliferative properties of the compounds were investigated using molecular docking experiments. Each compound’s best-docked poses, binding affinity, and receptor-ligand interaction were evaluated. Compounds’ molecular weights, logPs, TPSAs, abilities to pass the blood-brain barrier, GI absorption qualities, and CYPP450 inhibition have been given. When the activities of these molecules were examined in vitro, for the A-549 cell line, hydrazone **1e** had the minimum IC_50_ value of 13.39 μM. For the MDA-MB-231 cell line, oxadiazole **2l** demonstrated the lowest IC_50_ value, with 22.73 μM. For PC-3, hydrazone **1d** showed the lowest C50 value of 9.38 μM. The three most promising compounds were determined as compounds **1e**, **1d**, and **2a** based on their minimum IC_50_ values, and an additional scratch assay was performed for A-549 and MDA-MB-231 cells, which have high migration capacity, for the three most potent molecules; it was determined that these molecules did not show a significant antimetastatic effect.

## 1. Introduction

Cancer is one of the most difficult diseases to which humans are exposed, as well as one of the leading causes of mortality around the world. Lung, breast, and prostate cancer are the most frequently seen cancer types [1]. Although progress has been made at an incredible pace in the fight against cancer, unfortunately, the need for more potent and selective treatment methods with fewer side effects has not been fully met yet. Millions of people worldwide will benefit greatly from the discovery of more specific, target-based, and therefore less adverse, treatments for many different types of cancer. To that end, drug research and development studies against cancer are being conducted with great care in our country and worldwide.

Hydrazones are chemical compounds with a structure similar to aldehydes and ketones. They can be formed when the NNH_2_ group replaces the oxygen in aldehydes or ketones. Hydrazone derivatives have a broad range of pharmaceutical activities and are already being used in the market as anticancer, antiproliferative, antimicrobial, anti-inflammatory, and antiviral agents [2,3,4,5,6,7,8,9,10,11].

Oxadiazoles are aromatic, heterocyclic chemical compounds. They have four isomers: 1,2,3 oxadiazoles, 1,2,4 oxadiazoles, 1,2,5 oxadiazoles, and 1,3,4 oxadiazoles. In particular, 1,3,4 oxadiazoles possess antibacterial, anti-inflammatory antioxidant, anticancer, analgesic, and antiviral properties, and they have a broad range of applications [12,13,14,15,16,17,18,19,20].

**Figure 1:**
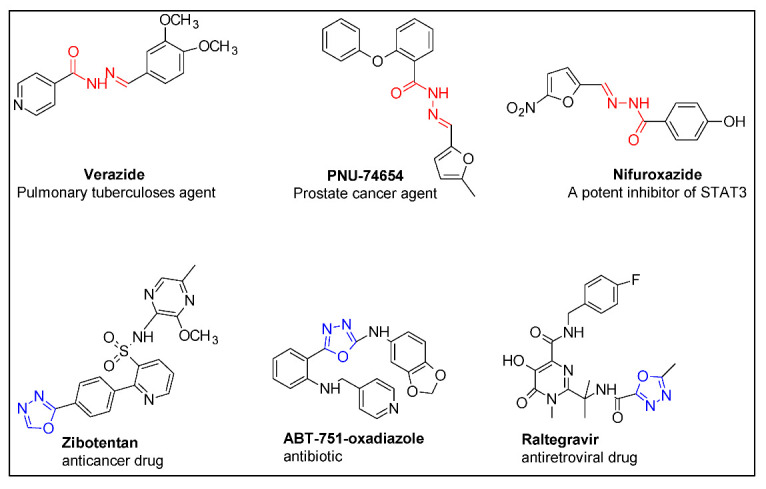
Structures of some hydrazones and 1,3,4-oxadiazoles used as pharmaceutical drugs in the clinic.

As an important class of medicinal chemistry, many of the differently substituted acyl hydrazones [21,22,23,24,25,26] and 1,3,4 oxadiazole derivatives [27,28,29,30,31,32] are already in the structures of some agents that are being used against cancer. The structures of some of those agents are shown in Figure 1 below.

**Scheme 1:**
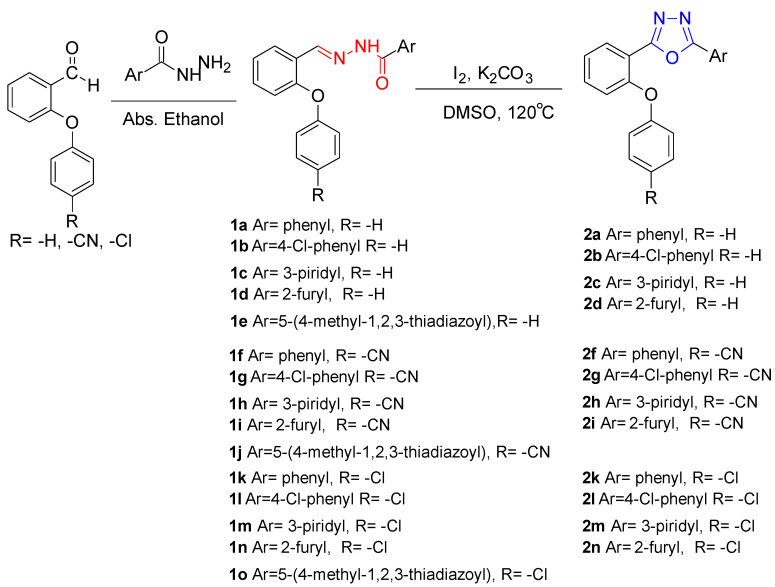
Synthesis of *N* −acyl hydrazones and 2,5 −substituted 1,3,4 −oxadiazoles.

In our previous study [33], we synthesized new *N*-acyl hydrazones **1a-o**, and 2,5-substituted 1,3,4-oxadiazoles **2a-d**, **2f-i**, and **2k-n**, encouraged by the great pharmaceutical potentials of acyl hydrazones and 1,3,4-oxadiazoles. The synthesis route is shown in Scheme 1 below.

In this study, cell lines representing lung, breast, and prostate cancers, which are among the most common cancer types, were selected to determine the anticancer activities of the N-acyl hydrazones and 1,3,4-oxadiazoles shown in Scheme 1. The A549 cell line was selected for lung cancer, MDA-MB-231, a triple-negative cell line was selected for breast cancer, and finally, the PC-3 cell line was selected for prostate cancer. The MRC-5 lung fibroblast cell line was also included in the study in order to see the damage the molecules would cause to non-cancerous tissues.

According to the results obtained, an additional scratch assay was performed for the three molecules that were found to have the most effect, and whether they were effective against metastasis was also investigated.

When biological activity analyses and docking studies are evaluated together, further studies can be carried out based on molecules that will be determined to be effective against cancer. In addition, other molecules can be designed and synthesized based on these molecules, and improvements can be made based on the results obtained.

## 2. Results

### 2.1. Biologic Activities

#### 2.1.1. In Vitro Cell Viability Assays

To determine the effect of N-acyl hydrazones and 2,5 substituted 1,3,4-oxadiazole derivatives, a dose-response curve was produced for each and every molecule based on the viability of A549 (lung carcinoma), PC-3 (prostate adenocarcinoma), and MDA-MB-231(breast adenocarcinoma) cancerous cell lines by using the Cell Titer Glo assay. The MRC-5 (lung fibroblast) cell line was used as the non-cancerous control group. For 48 h, the cytotoxic effects of novel derivatives on all of the cell lines were investigated in a dose-dependent manner.

The results are shown as IC_50_ values in Table 1 below.

As can be seen in Table 1, different substances displayed varying IC_50_ values in each cell line. Hydrazone **1e** showed the best activity for the A-549 cell line, with an IC_50_ value of 13.39 µM. The molecule with the best activity for the MDA-MB-231 cell line was oxadiazole **2l**, with an IC_50_ value of 22.73 µM. For the PC-3 cell line, the most effective molecule was hydrazone **1d**, with an IC_50_ value of 9.389 µM.

When the activities of these molecules on other lines are examined, it is seen that hydrazone **1e**, which is effective on lung cancer, has an IC_50_ of 18.09 µM in the PC-3 cell line and 108.3 µM in the MDA-MB-231 cell line. Another active molecule, hydrazone **1d**, has an IC_50_ of 49.79 µM in the A549 cell line and 31.49 µM in the MDA-MB-231 cell line. Finally, oxadiazole **2l**, which is the most effective among 27 molecules on breast cancer, has an IC_50_ of 38.42 µM on the PC-3 cell line and 36.26 µM on the A549 cell line. The concentrations of these substances on the MRC-5 fibroblast cells used in this study as a control group were found to be 44.66 µM for **1d**, 86.96 µM for **1e**, and 51.87 µM for **2l**.

When N-acyl hydrazones and 2,5-substituted 1,3,4-oxadiazoles are compared based on their activity on cell lines specifically, seven N-acyl hydrazones (**1a**, **1b**, **1d**, **1f**, **1g**, **1h**, and **1m**) on A549 appear to have lower IC_50_ values than their respective 1,3,4,-oxadiazoles. Based on cell viability results, it is possible to say that hydrazone derivatives are more effective on A549 cell lines than oxadiazole derivatives. Contrary to A549, oxadiazoles were found to be more effective on the MDA-MB-231 cell line than their respective N-acyl hydrazones. Only four N-acyl hydrazones (**1c**, **1g**, **1k**, and **1m)** were able to act at lower concentrations than their respective 1,3,4-oxadiazoles in the MDA-MB-231 cell line. Finally, in the PC-3 cell line, similar to the A549 cell line, N-acyl hydrazones are more effective than 1,3,4-oxadiazoles. Only four oxadiazoles (**2f**, **2h**, **2i**, and **2n**) managed to have lower IC_50_ values than their respective hydrazones. When the effects of synthesized compounds on the non-cancerous MRC-5 cell line were examined, it was determined that seven oxadiazoles (**2c**, **2d**, **2g**, **2h**, **2i**, **2k**, **2n**) caused a more destructive effect than their respective hydrazones. When all lines and molecules are considered, **2k** and **2n** oxadiazoles are found to be more active than hydrazones in all cancerous cell lines and also in the control group. For the remaining molecules, it is not possible to generalize the results that are valid for all of the cell lines since activities vary based on the lines.

**Figure 2:**
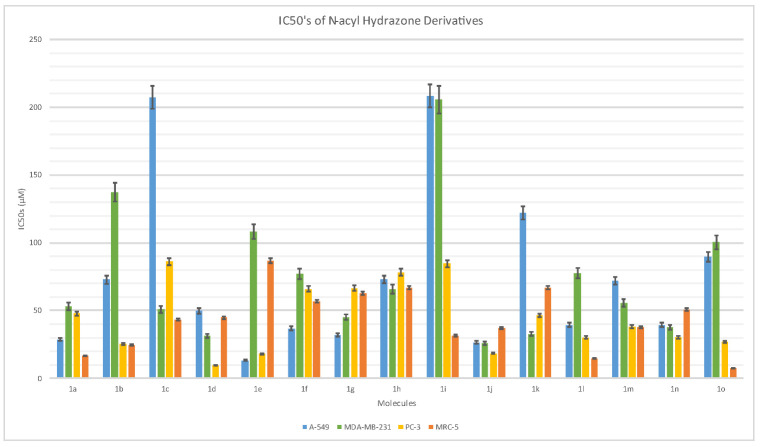
IC50 values of N-acyl hydrazones on A549, MDA-MB-231, and PC3 cell lines and MRC5 healthy human cells. Cells were treated with increasing dosages of (25–300 M) compounds. Control cells were treated with an equal amount of DMSO. Error bars show SEM.

**Figure 3:**
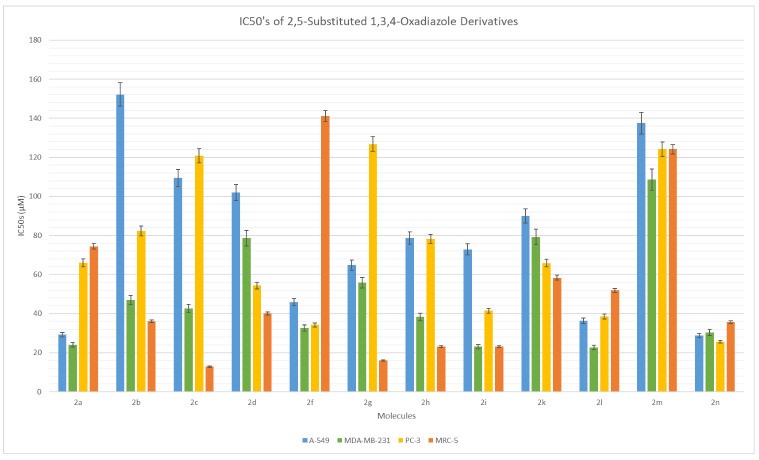
IC50 values of 2,5 substituted 1,3,4-oxadiazoles on A549, MDA-MB-231, and PC3 cell lines and MRC5 healthy human cells. Cells were treated with increasing dosages of (25–300 M) compounds. Control cells were treated with an equal amount of DMSO. Error bars show SEM.

Because of the large number of molecules and the use of multiple cell lines in the research, it is critical to portray the data in Table 1 over graphs. The effects of N-acyl hydrazones on all cell lines are depicted in Figure 2, and 2,5-substituted 1,3,4-oxadiazoles are depicted in Figure 3 as bar graphs.

#### 2.1.2. Scratch Assay

Because A-549 and MDA-MB-231 are two of the most metastatic cell lines tested in this study, a scratch assay was performed to see if the three most active compounds (**2a**, **1d**, **1e**) had any antimetastatic effect on these lines.

**Figure 4:**
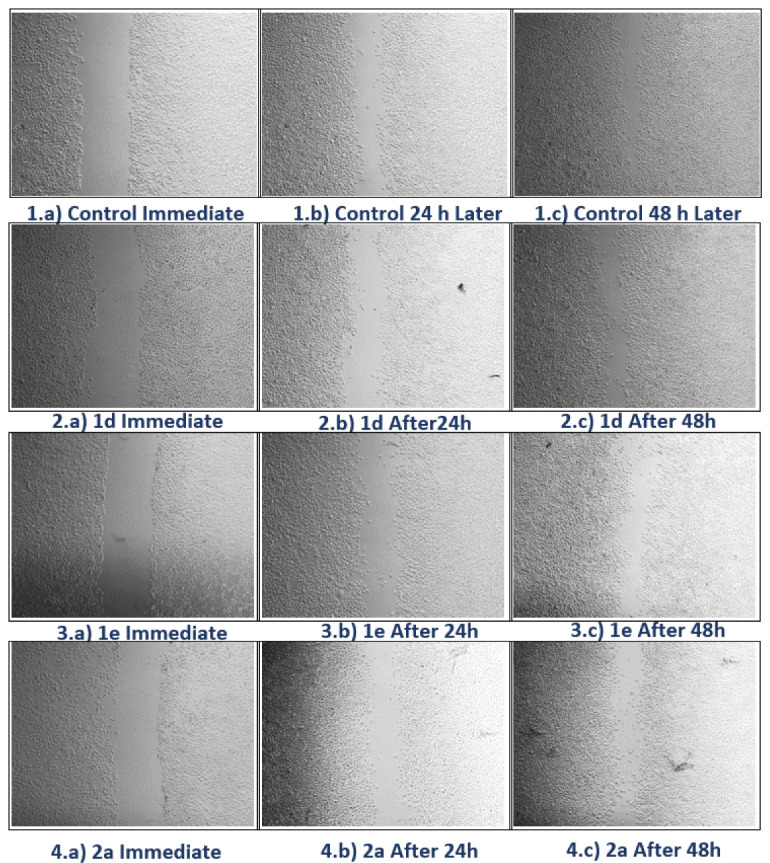
Analysis of cell migration by scratch assay for molecules **1d**, **1e**, and **2a** on A549 cell line. Images were taken at 0 h, and 24 h, 48 h after incubation. Concentrations were 55 µM for **1d**, 15 µM for **1e**, and 15 µM for **2a**. Control groups were treated with an equal amount of DMSO solvent.

**Figure 5:**
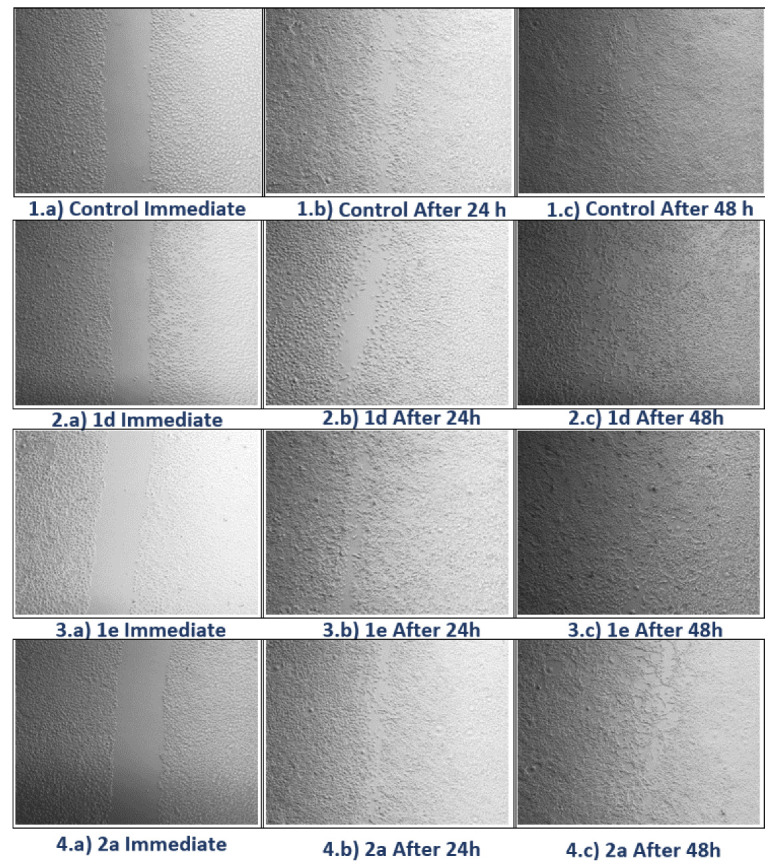
Analysis of cell migration by scratch assay for molecules **1d**, **1e**, and **2a** on MDA-MB-231 cell line. Images were taken at 0 h, and 24 h, 48 h after incubation. Concentrations were 40 µM for **1d**, 120 µM for **1e**, and 30 µM for **2a.** Control groups were treated with an equal amount of DMSO solvent.

The dosages for MDA-MB-231 were 40 µM for **1d**, 120 µM for **1e**, and 30 µM for **2a**. The dosages for A-549 were 55 µM for **1d**, 15 µM for **1e**, and 15 µM for **2a**. Three photos were taken: one immediately after the application, one 24 h later, and one 48 h later. The images of each time stamp for all three molecules can be seen in Figure 4 for the A549 cell line and Figure 5 for the MDA-MB-231 cell line.

When the wound healing rates were compared, it was discovered that the added molecules did not affect the wound healing rate. To make comparisons, each application was performed in triplicate for all three molecules; the averages and standard deviations were calculated, and the p values were calculated by considering the sample sizes.

### 2.2. Molecular Docking Studies

The binding capacities of these novel compounds were investigated with molecular docking studies, and the best-docked poses of the molecules were thoroughly evaluated. The best binding affinity and receptor-ligand interaction of every compound were assessed, and well-established good interactions of compounds within the receptor’s active pocket of the target receptor proteins were demonstrated in Table 2, Table 3 and Table 4. We chose our targets based on the previous research of similar structures possessing hydrazone derivatives. We decided to look for possible binding motifs for Janus kinase 2 to investigate their anticancer activity on breast cancer, phosphoinositide-dependent kinase-1 to investigate their activity on prostate cancer, and human anaplastic lymphoma kinase to investigate their activity on lung cancer [34,35,36]. We compared our results with the often-used anticancer medicines doxorubicin, crizotinib, and tamoxifen.

Molecular docking studies were carried out to provide a theoretical viewpoint on potential molecular interactions between **1a–1o** and **2a–2n** series molecules and target proteins. Energy minimization from docking calculation results was used to determine theoretical binding affinities. The Autodock Vina software suite was used to perform molecular docking computations, energy minimization, and molecular visualization of docking data. The Chem Draw sketch program was used to prepare **1a–1o** and **2a–2n** series and model inhibitor compounds for molecular docking. The Chem 3D suit program was used to draw and edit the unique **1a–1o** and **2a–2n** series chemicals in the SD file format prior to the docking process. These molecular structures were protonated and charged, and conformation minimization using the root mean square gradient was conducted.

The target proteins’ X-ray crystal structures as three-dimensional coordinates were retrieved from the Protein Data Bank of the Research Collaboratory for Structural Bioinformatics (RCSB) (https://www.rcsb.org accessed on 1 October 2022). Structural defects in the target proteins were eliminated with the Autodock Vina suite software. Default parameters were used while docking calculations were in progress (temperature of 300 Kelvin, pH 7, solvent 0.1 M, electrostatic energy cutoff 15 A). The final molecular docking score results were calculated using the average score of the top 10 final docking postures determined by the binding minimum energy (kcal/mol) for each chemical [37,38].

The compounds demonstrated the following binding free energies towards 1Z5M, 2XP2, and 3KRR: −8.89 to −11.47 Kcal/mol for 1Z5M, −8.47 to −9.81 Kcal/mol for 2XP2, and −9.19 to −11.11 Kcal/mol for 3KRR.

**Figure 6:**
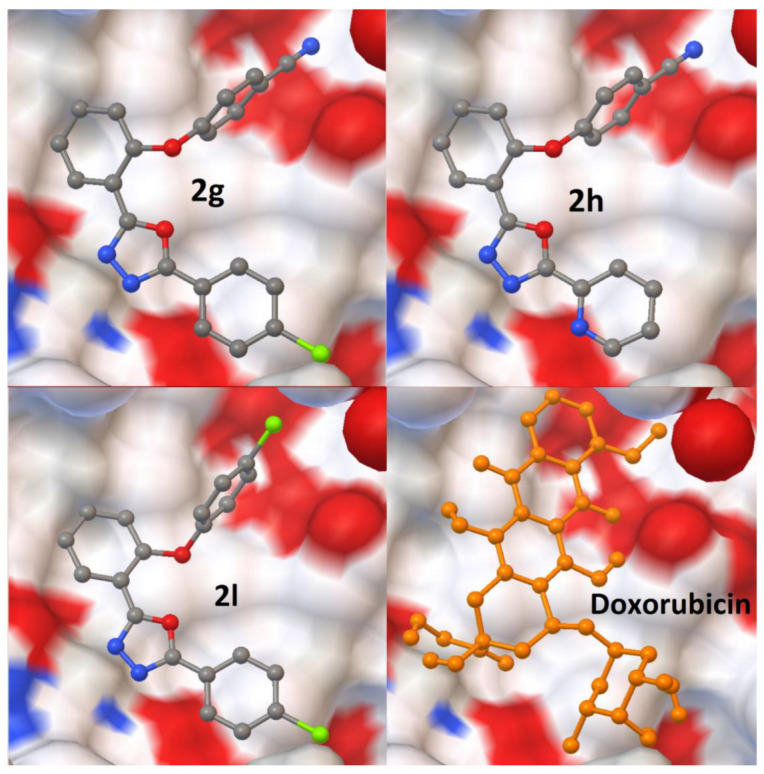
Interaction of the best-docked poses of compounds **4g**, **4h**, **4l**, and reference drug doxorubicin to 1Z5M target.

**Figure 7:**
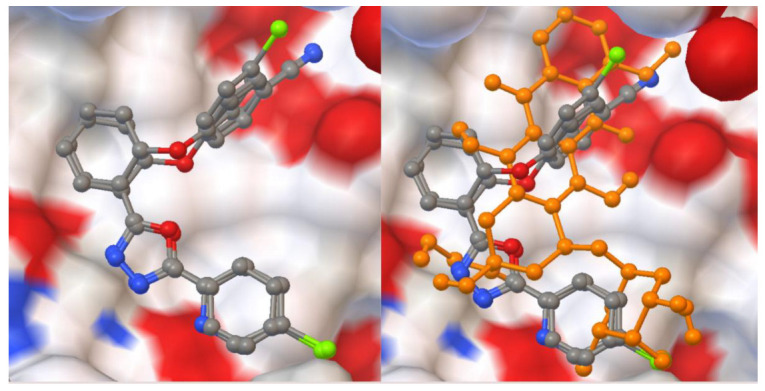
Superimposing poses of best-scored compounds with and without reference drug doxorubicin against prostate cancer.

**Figure 8:**
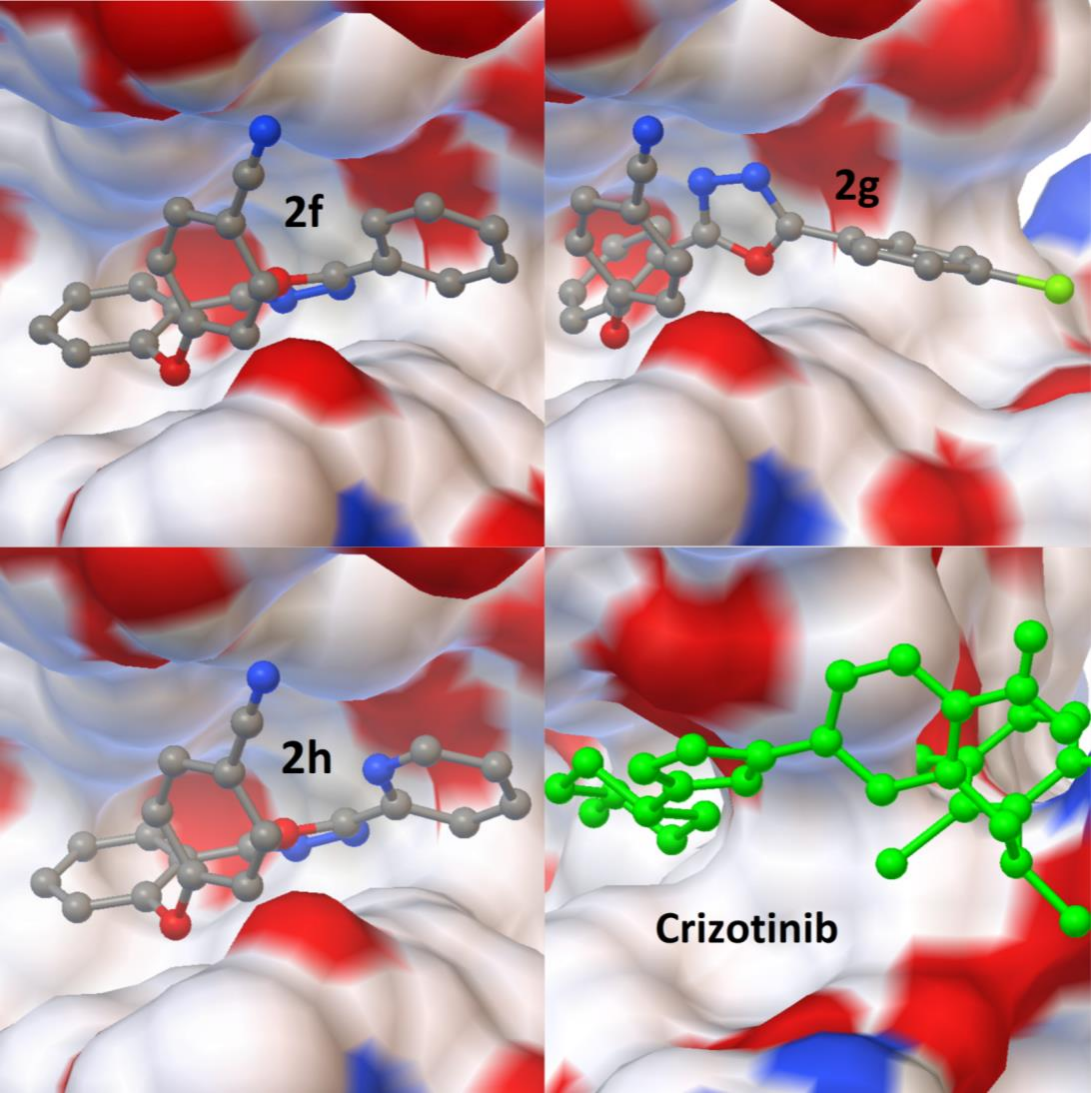
Interaction of the best-docked poses of compounds **4f**, **4g**, **4h** and reference drug crizotinib to 2XP2 target.

**Figure 9:**
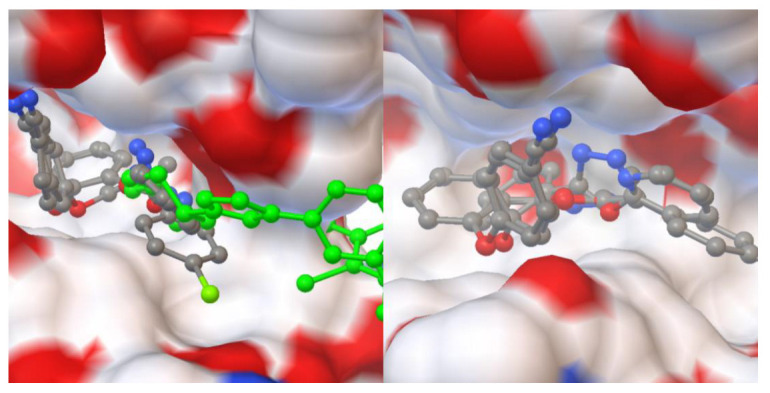
Superimposing poses of best scored compounds with and without reference drug crizotinib against lung cancer.

**Figure 10:**
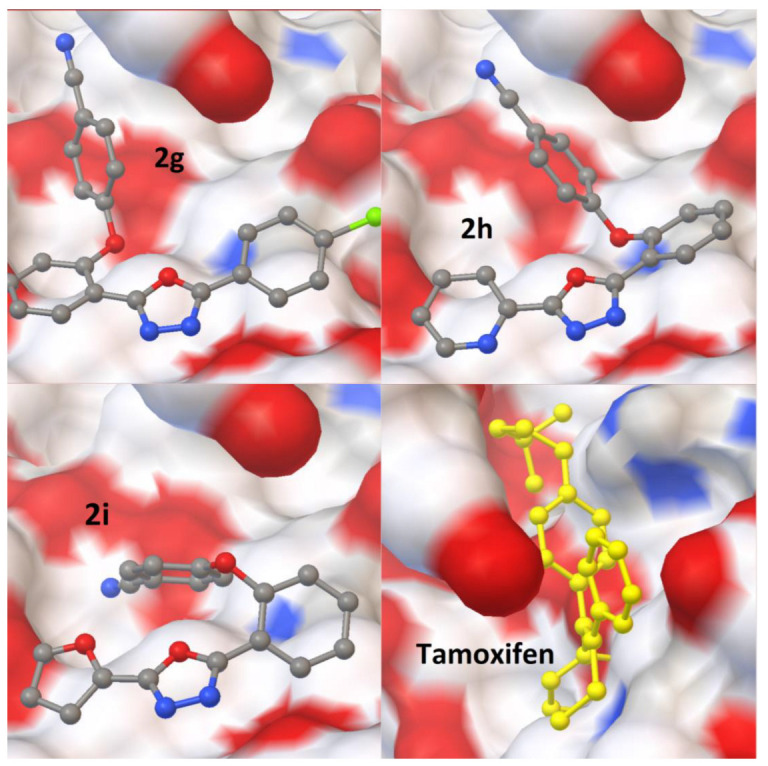
Interaction of the best-docked poses of compounds **4g**, **4h**, **4i** and reference drug tamoxifen to 3KRR target.

**Figure 11:**
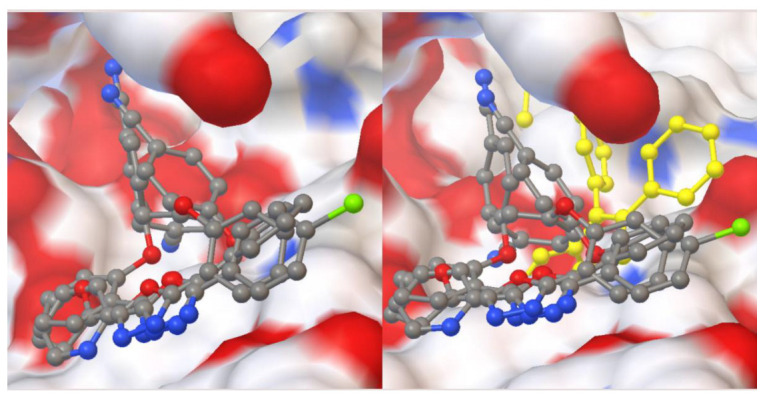
Superimposing poses of best scored compounds with and without reference drug tamoxifen against breast cancer.

As demonstrated in Figure 6, Figure 7, Figure 8, Figure 9, Figure 10 and Figure 11, molecules bound to the active site and overlapped with reference molecules. Our initial findings indicate that these compounds have reasonable ligand-receptor binding interactions. 

Compounds **2f**,**2g**, **2h**, **2i**, and **2l** have the lowest binding energy scores, with small RMSD scores in each target. Some of them created strong hydrogen bonds with similar amino acid residues, as indicated in the tables. Active conformations of each compound bonded active sites and overlapped with each other, as shown in the figures. These compounds predicted the best ligand-receptor binding interactions, according to the results.

We also calculated RMSD values between the native structures in solvent and docked structures as follows: 0.64 for 1Z5M, 0.78 for 2XP2, and 0.69 for 3KRR. All three re-docked reference structure conformations showed high similarity to native conformations.

### 2.3. Drug-like Properties

To better understand the structure-activity relationships of compounds, drug-likeness rankings have been calculated through the usage of the Swiss ADME Calculation program. All of the compounds’ molecular weights, logP values, TPSAs, their abilities to pass through the blood-brain barrier, GI absorption properties, and CYP450 subtype inhibition have been provided in Table 5. Almost all substances have been identified to have relatively low values that allow them to pass through the lipid barriers. Most substances have lipophilicity values of less than four.

## 3. Discussion

The anticancer activities of fifteen N-acyl hydrazones and twelve 2,5-substituted 1,3,4-oxadiazoles were investigated in this study by calculating IC-50 values on A-549 (human lung cancer epithelial cells), MDA-MB-231 (human breast carcinoma), and PC-3 (human prostatic carcinoma) cell lines.

The cytotoxic activities of the synthesized compounds were studied at concentrations of 300 µM, 200 µM, 100 µM, 50 µM, and 25 µM. When all the data obtained during the study were reviewed together, the most potent compounds among the twenty-seven molecules tested were determined to be hydrazones **1d** and **1e** and oxadiazole **2a**. While making this evaluation, the effects of the molecules on all the cancerous cell lines and their toxicity on normal cells were taken into consideration.

A scratch assay was performed for the three most promising compounds, 1d, 1e, and 2a, based on the data obtained from these scans. In this way, it was anticipated to see if these molecules have an antimetastatic effect.

It was discovered throughout the study that the hydrazones used in the production of oxadiazoles did not exhibit a consistent difference in biological activity when compared to their counterparts in any of the cell lines. According to research, molecules respond differently in different types of cancer. As a result, it is not possible to draw a firm assumption that applies to all cell lines.

If some modifications are made to the chemical structures of potent compounds to reduce their toxicity and boost their activity against cancer, it may be possible to re-evaluate selected candidate compounds as more promising anticancer agents.

The most potent molecule, hydrazone **1e**, demonstrated a particularly selective effect in PC-3 and A-549 cell lines. This molecule also has a low toxicity level on the MRC-5 cell line. This hydrazone’s structure includes a thiadiazol heterocyclic aromatic ring. Thiadiazols are frequently studied structures by medicinal chemists. This heterocyclic ring has a mesoionic character, which allows candidate molecules with this ring in their structures to permeate the cell membrane more effectively and have a stronger connection with biological targets. Therefore, it would be wise to investigate this thoroughly in other cancer cell lines and further study the mechanism of action in biological systems.

MDA-MB-231 cells are triple-negative cells that do not express some hormone receptors for HER2, estrogen, and progesterone on their surfaces, so this might be a reason why **1e** has a lower impact on them than A-549 and PC-3 cells since the possible target might be missing. Hence, it is believed that the heterocyclic structure of thiadiazol in the molecule structure might influence receptor binding capacity and lower the effectiveness of the molecule. In a study conducted by Du et al., activities of 1,3,4-oxadiazole-thioether derivatives on various cancer lines were examined. Based on the obtained results, it was reported that the activity of their most effective derivative (compound **3**) against breast cancer-representing cell line MCF-7 (18.3 µM) was less than that of the liver cancer-representing cell line HepG2 (0.7 µM) [39].

In almost all of the 1,3,4 oxadiazole derivatives included in another study conducted by Ashok and Vanaja, the activities on the lung cancer-representing cell line A549 were better than MCF-7, similar to our study [40]. Another study comparing the level of activity in the MCF-7 cell line with that of A-549 was reported by Polothi et al. Additionally, in this study, it was observed that the activity levels of 1,3,4 oxadiazole derivatives on A549 were better than MCF-7 [41].

More active molecules might be generated by synthesizing and analyzing the new derivatives of this compound in the future.

## 4. Materials and Methods

### 4.1. Cell Culture

In this study, four adherent cell lines were used: three from different cancer types and one from a regular fibroblast. The cancerous cells were human breast carcinoma (MDA-MB-231, ATCC code: HTB-26), human prostatic carcinoma (PC-3, ATCC code: CRL-1435), and human lung cancer epithelial cells (A549, ATCC code: CCL-185), while the fibroblasts in the control group were human fetal lung (MRC-5, ATCC code: CCL-171) cells. All cells were checked every few weeks for various sources of contamination.

MDA-MB-213, MRC-5, and PC-3 cell lines were cultured in T75 flasks with high glucose DMEM medium (Pan Biotech, Aidenbach, Germany), and A549 cells were cultured with RPMI-1640 (Pan Biotech) containing 10% fetal bovine serum (Gibco, Waltham, MA, USA), 1% penicillin-streptomycin (Gibco), and 1% l-glutamine (Gibco) at 37 °C with 5% CO_2_. All the cells were removed from the flask with Trypsin/EDTA 0.25% (Gibco) when they reached 80% density.

### 4.2. Cell Viability Assay

To test the effect of different doses on cells, all types of cells were seeded into 96-well plates at a density of eight thousand cells in 100 µL volume per well. Cells were incubated at 37 °C with 5% CO_2_ for 25 h_._ After 24 h in the incubator, the medium on top of each well was discharged. All of the cells were treated with 25, 50, 100, 200, or 300 µM of fifteen N-acyl hydrazones and twelve 2,5-substituted 1,3,4-oxadiazole in a dose-increasing manner. Molecules were studied in triplicate for each concentration. To assess the viability of non-treated cells, a control group was placed in the setup with only cells and DMSO control treatments without any candidate molecules introduced. Plates were incubated for 48 h, and Cell Titer Glo reagent (Promega) was added on top of each well. Cells were then evaluated in terms of viability with the Cell Titer-Glo Luminescent Cell Viability Assay. Luminescence signals were detected with the SpectraMax i3x Multimode Detection Platform to obtain the cell viability percentages.

### 4.3. Scratch Assay

MDA-MB-231 and A-549 cells were seeded for the scratch assay. A total of 100.000 cells were cultured in 1.5 mL complete medium per well on a 6-well plate, as detailed above. The 6-well plates were then incubated for 24 h at 37 °C. After 24 h, accumulated cells were scraped out with the tip of a 200 µL pipette. After discarding the media on top of the cells, the cells were treated for 48 h with three molecules: **1d**, **1e**, and **2a**.

The dosages for the MDA-MB-231 cell line were 40 µM for **1d**, 120 µM for **1e**, and 30 µM for **2a**. The dosages for the A-549 cell line were 55 µM for **1d**, 15 µM for **1e**, and 15 µM for **2a**.

The cell states were evaluated under the microscope, and markings were made to obtain photos from the same location during the follow-up. Images of the determined reference points were captured under the microscope immediately after treatment, 24 h later, and 48 h later.

### 4.4. Statistical Analysis

Experiments were carried out in three separate sets, each with its own set of results, and the findings were represented as mean standard errors. Statistical comparisons were made using Student’s *t*-test, which claims equal variance. At *p* < 0.05, the differences were accepted as statistically significant. The data were presented as a standard error of the mean (SEM).

### 4.5. Molecular Docking Studies and Drug-like Properties

Molecular docking analyses were implemented to offer a theoretical perspective on potential molecular encounters between hydrazone compounds of **1a**–**1o** and oxadiazole compounds of **2a**–**2n** and related target proteins. Theoretical binding affinities were determined by using energy minimization from docking calculation outcomes. The Autodock Vina software suite was used to perform molecular docking computations, energy minimization, and molecular visualization of docking data. The Chem Draw sketch program was used to prepare **1a**–**1o** and **2a**–**2n** series and model inhibitor compounds for molecular docking. The Chem 3D suit program was used to draw and edit the unique **1a**–**1o** and **2a**–**2n** series chemicals in the SD file format before the docking process. These conformations were protonated and polarized, and conformation minimization was performed using the root-mean-square gradient.

The X-ray crystal structures of the target proteins were obtained as three-dimensional coordinates from the Protein Data Bank of the Research Collaboratory for Structural Bioinformatics (RCSB). A structure with the PDB ID of 1Z5M for PC-3, 2XP2 for A-549, and 3KRR for MDA-MB-231 were selected as the crystal structure model corresponding to this target protein for use in docking calculations. The Autodock Vina suite program was used to remove structural flaws in these target proteins. Default parameters were used while docking calculations were in progress (temperature of 300 K, pH of 7, solvent concentration of 0.1 M, and electrostatic energy cutoff of 15 A). The average score of the top ten final docking postures defined by the binding minimum energy (kcal/mol) for each molecule was used to generate the final molecular docking score values [37,38]. 

In order to gain a better understanding of compound structure-activity connections, drug-likeness rankings were determined using the Swiss ADME Calculation program.

## 5. Conclusions

In this study, previously synthesized novel 2-arenoxybenzaldehyde N-acyl Hydrazone and 1,3,4-Oxadiazole derivatives were evaluated for their anticancer activities in different cancer cells. Compounds **1d** and **1e** contain hydrazone moieties, and **2a**, which contains a 1,3,4-oxadiazole moiety, yielded the most promising findings among all molecules. Molecular modeling investigations on the synthesized compounds revealed further structural and dynamic information about their possible target proteins. The activities of the compounds synthesized in this study are not a better alternative than other available molecules on the market.

However, the findings suggest that it is possible to synthesize several derivatives that might be employed in future research to examine different cancer pathways. More research into these novel derivatives and their anticancer effects may offer more insights into potential therapeutic applications.

## Figures and Tables

**Table 1:** A549, MDA-MB-231, PC-3, and MRC-5 cells were treated with N-acyl hydrazones and 2,5 substituted 1,3,4-oxadiazoles. The effect of the molecules on cell viability is expressed as IC_50_ values (µM). Each experiment was performed in triplicate. Data are expressed as means.

Molecules *	A549	MDA-MB-231	PC-3	MRC-5
**1a**	28.65	53.02	47.75	16.89
**2a**	29.28	24.02	65.96	74.45
**1b**	72.81	137.50	25.27	24.44
**2b**	152.20	46.92	82.23	36.02
**1c**	207.50	50.84	86.02	43.46
**2c**	109.40	42.62	120.90	12.82
**1d**	49.79	31.46	9.39	44.66
**2d**	102.10	78.60	54.31	40.07
**1e**	13.39	108.30	18.09	86.96
**1f**	36.90	77.20	65.91	56.82
**2f**	45.79	32.66	34.16	141.10
**1g**	31.84	45.08	66.53	62.75
**2g**	64.78	55.74	126.80	16.00
**1h**	73.04	65.80	78.34	66.87
**2h**	78.58	38.20	78.22	23.07
**1i**	208.50	20.70	84.56	31.66
**2i**	72.85	23.07	41.37	23.07
**1j**	26.56	25.90	18.39	37.06
**1k**	121.90	32.69	46.35	66.87
**2k**	89.98	79.20	65.83	58.34
**1l**	39.43	77.70	30.20	14.55
**2l**	36.26	22.73	38.42	51.87
**1m**	71.77	55.51	38.12	37.93
**2m**	137.47	108.50	124.10	124.10
**1n**	39.36	37.85	30.39	50.55
**2n**	28.84	30.37	25.60	35.59
**1o**	89.65	100.40	27.03	7.78

* The numbers 1 and 2 are given next to the molecules in the table to indicate whether they are hydrazones (1) or oxadiazoles (2), as well as letters to understand which oxadiazole is produced from which hydrazone. As a result, it is apparent from the table that, for instance, oxadiazole, encoded as **2a**, was synthesized utilizing the hydrazone encoded as **1a**.

**Table 2:** Docking results of phosphoinositide-dependent kinase-1 (1Z5M).

Comp No.	Structures	Docked Amino Acid Residues (vdW Interactions)	Energy Score	RMSD Value	H Bond (Distance Å)
**1a**	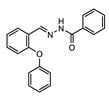	GLY89, ALA162, LEU212	−9.31	0.96	O of Carbonyl with H of NH of TYR161 (1.772)
**1b**	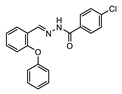	TYR161, ALA162, GLY165	−9.66	1.00	None
**1c**	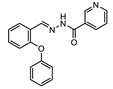	VAL96, ALA109, ALA162	−9.47	1.62	H of NH with O of Carbonyl of GLU166 (2.115)
**1d**	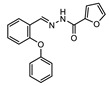	LEU159, SER160, LEU212	−9.46	0.58	O of Carbonyl with H of NH of ALA162 (2.241)
**1e**	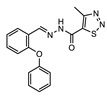	VAL96, ALA109, SER160	−8.89	0.33	None
**1f**	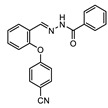	VAL96, ALA109, SER160	−9.63	1.50	H of NH with O of Carbonyl of GLU166 (2.149)
**1g**	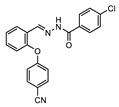	LYS86, ALA162, GLU166	−8.92	1.89	None
**1h**	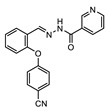	LEU88, GLU166, LEU212	−8.97	1.64	None
**1i**	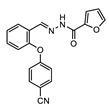	VAL96, VAL143, THR222	−10.50	0.50	H of OH with O of Carbonyl of SER160 (2.064)
**1j**	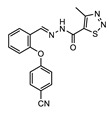	LEU88, LYS111, GLU166	−9.87	0.38	H of imine with H of OH of THR222 (2.230)
**1k**	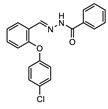	ALA109, ALA162, THR222	−9.65	1.86	None
**1l**	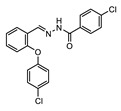	VAL96, ALA109, LEU212	−9.64	1.19	H of NH with O of Carbonyl of GLU166 (2.207)
**1m**	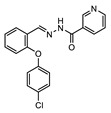	ALA109, ALA162, THR222	−9.54	1.99	H of NH with O of Carbonyl of GLU166 (2.168)
**1n**	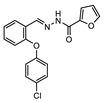	GLY89, LYS111, SER160,	−9.76	0.59	O of Carbonyl with H of NH of ALA162 (2.220)
**1o**	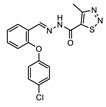	LEU88, GLY165, GLU166	−9.61	0.93	H of NH with O of Carbonyl of GLU209 (2.072)
**2a**	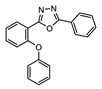	VAL96, LEU159, THR222	−10.47	0.12	N of Oxazole with H of NH of TYR161 (2.023)
**2b**	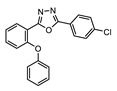	LEU159, ALA162, THR222	10.78	0.15	N of Oxazole with H of NH of TYR161 (1.933)
**2c**	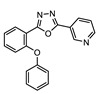	VAL96, LEU159, THR222	−10.45	0.86	N of Oxazole with H of NH of ALA162 (1.912)
**2d**	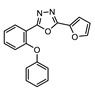	VAL96, LEU159, THR222	−10.31	0.17	N of Oxazole with H of NH of ALA162 (2.249)
**2f**	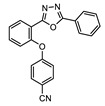	VAL96, LEU159, THR222	−11.13	0.23	N of Oxazole with H of NH of ALA162 (1.964)
**2g**	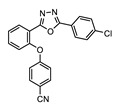	LEU159, TYR161, THR222	−11.47	0.09	N of Oxazole with H of NH of ALA162 (1.948)
**2h**	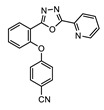	VAL96, LEU159, THR222	−11.24	0.11	N of Oxazole with H of NH of ALA162 (1.687)
**2i**	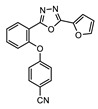	VAL96, LEU159, THR222	−11.07	0.27	N of Oxazole with H of NH of ALA162 (1.811)
**2k**	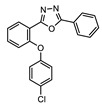	VAL96, LEU159, THR222	−10.84	0.26	N of Oxazole with H of NH of ALA162 (2.004)
**2l**	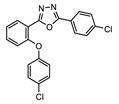	VAL96, LEU159, THR222	−11.22	0.11	N of Oxazole with H of NH of ALA162 (1.966)
**2m**	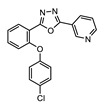	VAL96, LEU159, THR222	−10.88	0.13	N of Oxazole with H of NH of ALA162 (1.967)
**2n**	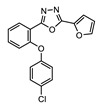	VAL96, LEU159, THR222	−10.79	0.26	N of Oxazole with H of NH of ALA162 (1.957)
**Doxorubicin**	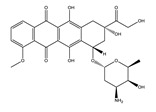	VAL96, ALA162, THR222	−12.92	0.89	H of OH with O of Carbonyl of GLU166 (2.035)

**Table 3:** Docking results of human anaplastic lymphoma kinase (2XP2).

Comp No.	Structures	Docked Amino Acid Residues (vdW Interactions)	Energy Score	RMSD Value	H Bond (Distance Å)
**1a**	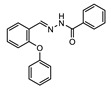	LEU1198, GLY1202, LEU1256	−8.72	1.93	O of Carbonyl with H of NH of MET1199 (1.932)
**1b**	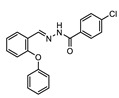	LEU1198, GLY1202, LEU1256	−9.23	0.75	None
**1c**	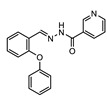	LEU1198, GLY1202, LEU1256	−9.17	0.84	O of Carbonyl with H of NH of MET1199 (2.099)
**1d**	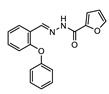	MET1199, LEU1256, LEU1196,	−9.14	0.45	H of NH with O of Carbonyl of GLU1197 (2.075)
**1e**	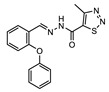	LEU1196, LEU1198, LEU1256	−8.65	1.91	N of Thiazole with H of NH of MET1199 (2.221)
**1f**	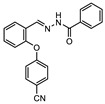	LEU1196, LEU1198, LEU1256	−9.18	0.41	N of Thiazole with H of NH of MET1199 (2.036)
**1g**	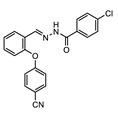	ALA1148, LEU1198, ASP1203	−8.99	1.87	N of Thiazole with H of NH of MET1199 (2.158)
**1h**	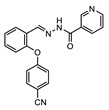	LEU1196, LEU1198, LEU1256	−8.71	1.95	N of Thiazole with H of NH of MET1199 (2.050)
**1i**	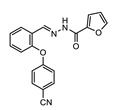	LEU1122, GLU1197, LEU1256	−9.65	0.28	N of Thiazole with H of NH of MET1199 (2.041)
**1j**	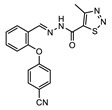	GLU1197, MET1199, ASP1203	−8.70	0.17	O of Carbonyl with H of NH of LYS1150 (2.056)
**1k**	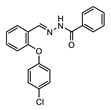	LEU1122, MET1199, LEU1256	−8.47	0.77	O of Carbonyl with H of NH of LYS1150 (1.986)
**1l**	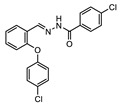	LEU1122, ALA1148, LEU1256	−9.18	0.34	H of NH with O of Carbonyl of MET1199 (2.122)
**1m**	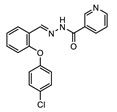	LEU1122, MET1199, LEU1256	−8.49	1.84	H of NH with O of Carbonyl of GLU1197 (2.143)
**1n**	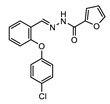	LEU1196, GLU1197, ASP1203	−9.07	0.81	H of NH with O of Carbonyl of MET1199 (1.895)
**1o**	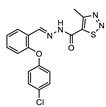	LEU1196, MET1199, LEU1256	−9.38	0.50	H of NH with O of Carbonyl of GLU1197 (2.117)
**2a**	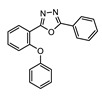	LEU1122, GLU1197, LEU1256	−8.81	0.21	N of Oxazole with H of NH of mMET1199 (1.810)
**2b**	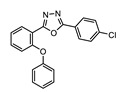	LEU1198, GLU1197, LEU1256	−9.25	0.13	N of Oxazole with H of NH of mMET1199 (1.742)
**2c**	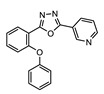	LEU1196, LEU1198, LEU1256	−8.89	0.30	N of Oxazole with H of NH of mMET1199 (1.758)
**2d**	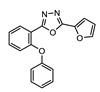	LEU1122, LEU2298, GLY1202	−8.73	1.13	N of Oxazole with H of NH of mMET1199 (1.758)
**2f**	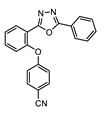	LEU1198, ASP1203, LEU1256	−9.72	0.34	N of Oxazole with H of NH of MET1199 (2.205)
**2g**	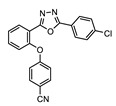	LEU1198, ASP1203, LEU1256	−9.75	1.34	N of Oxazole with H of NH of MET1199 (2.968)
**2h**	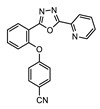	LEU1198, ASP1203, LEU1256	−9.81	0.11	N of Oxazole with H of NH of MET1199 (2.184)
**2i**	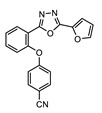	LEU1198, ASP1203, LEU1256	−9.42	0.77	N of Oxazole with H of NH of MET1199 (1.840)
**2k**	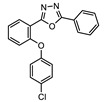	ALA1146, LEU1198, LEU1256	9.18	0.42	N of Oxazole with H of NH of MET1199 (1.791)
**2l**	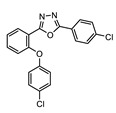	LEU1198, GLU1197, LEU1256	−8.74	1.54	N of Oxazole with H of NH of MET1199 (1.973)
**2m**	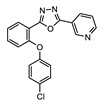	ALA1148, LEU1198, LEU1256	−9.26	0.43	N of Oxazole with H of NH of MET1199 (1.832)
**2n**	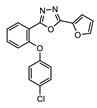	ALA1148, LEU1198, LEU1256	−9.18	0.12	N of Oxazole with H of NH of MET1199 (1.780)
**Crizotinib**	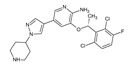	LEU1196, LEU1198, MET1199	−9.93	1.25	None

**Table 4:** Docking results of Janus kinase 2 (3KRR).

Comp No.	Structures	Docked Amino Acid Residues (vdW Interactions)	Energy Score	RMSD Value	H Bond (Distance Å)
**1a**	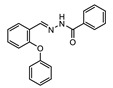	LEU855, LEU932, GLY993	−9.68	1.11	None
**1b**	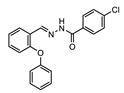	LEU855, LEU932, LEU983	−10.08	1.10	None
**1c**	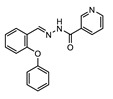	LEU855, LEU932, GLY993	−9.68	1.38	None
**1d**	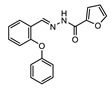	LEU855, LEU932, GLY993	−9.76	-0.29	None
**1e**	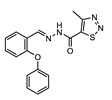	ALA880, LEU932, GLY993	−9.47	0.87	N of Thiazole with H of NH of LEU932 (2.066)
**1f**	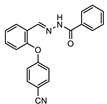	VAL863, ALA880, LEU932	−9.65	0.49	O of Carbonyl with H of NH of SER936 (2.089)
**1g**	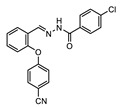	LEU855, LEU932, GLY993	−9.39	1.73	None
**1h**	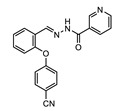	LEU855, LEU932, GLY993	−9.87	0.39	H of NH with O of Carbonyl of ASN961 (2.204)
**1i**	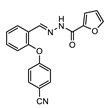	LEU855, LEU932, LEU983	−10.05	0.49	None
**1j**	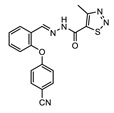	LEU855, LEU932, LEU983	−9.68	0.84	None
**1k**	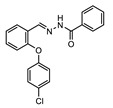	LEU855, GLY935, LEU983	−10.09	0.51	O of Carbonyl with H of NH of LEU932 (1.919)
**1l**	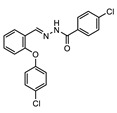	LEU855, GLY935, LEU983	−10.57	0.93	O of Carbonyl with H of NH of LEU932 (1.971)
**1m**	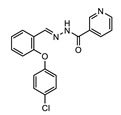	GLY856, GLY861, GLY993	−10.05	1.40	H of NH with O of Carbonyl of ASN961 (2.215)
**1n**	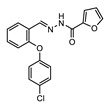	LEU855, TYR931, LEU983	−10.02	0.49	H of NH with O of Carbonyl of GLU930 (2.227)
**1o**	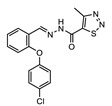	LEU932, SER936, LEU983	−9.19	0.19	None
**2a**	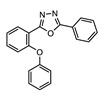	LEU855, MET929, LEU983	−10.39	0.14	N of Oxadiazole with H of NH of LEU932 (2.004)
**2b**	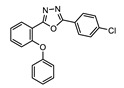	LEU855, MET929, LEU983	−10.70	0.11	N of Oxadiazole with H of NH of LEU932 (2.042)
**2c**	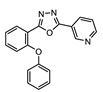	LEU855, MET929, LEU983	−10.23	0.40	N of Oxadiazole with H of NH of LEU932 (2.193)
**2d**	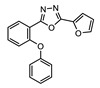	LEU855, MET929, LEU983	−10.22	0.11	N of Oxadiazole with H of NH of LEU932 (2.051)
**2f**	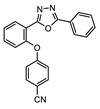	LEU932, SER936, LEU983	−10.83	0.16	None
**2g**	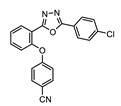	LEU855, LEU932, LEU983	−11.11	0.12	N of Oxadiazole with H of NH of LEU932 (1.988)
**2h**	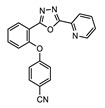	LEU855, LEU932, LEU983	−10.85	0.06	N of Oxadiazole with H of NH of LEU932 (1.974)
**2i**	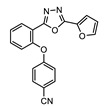	LEU855, LEU932, LEU983	−10.90	0.13	N of Oxadiazole with H of NH of LEU932 (2.078)
**2k**	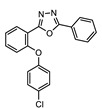	LEU855, LEU932, LEU983	−10.66	0.93	N of Oxadiazole with H of NH of LEU932 (1.849)
**2l**	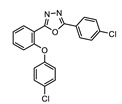	LEU855, LEU932, LEU983	−10.48	0.89	None
**2m**	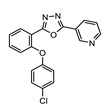	LEU855, LEU932, LEU983	−10.52	1.04	N of Oxadiazole with H of NH of LEU932 (1.855)
**2n**	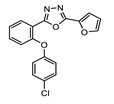	LEU855, LEU932, LEU983	−10.57	0.20	N of Oxadiazole with H of NH of LEU932 (2.159)
**Tamoxifen**	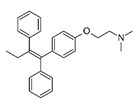	LEU855, LEU983, GLY993	−12.06	1.21	None

**Table 5:** Drug-like properties of **1a-1o**, **2a-2n comps.** calculated by the Swiss ADME online software program.

Comp. No	MW (g/mol) ^a^	LogP ^b^	TPSA ^c^	BBB ^d^	GI Abs. ^e^	Type of CYP Inh. ^f^	Rule of Five ^g^
**3a**	316.35	4.03	50.69	Yes	High	CYP1A2, CYP2C19, CYP2C9, CYP2D6	Yes
**3b**	350.80	4.58	50.69	Yes	High	CYP1A2, CYP2C19, CYP2C9	Yes
**3c**	317.34	3.13	63.58	Yes	High	CYP1A2, CYP2C19, CYP2C9, CYP2D6	Yes
**3d**	306.32	3.40	63.83	Yes	High	CYP1A2, CYP2C19, CYP2C9, CYP2D6	Yes
**3e**	338.38	3.19	104.71	Yes	High	CYP1A2, CYP2C19, CYP2C9	Yes
**3f**	341.36	3.87	74.48	Yes	High	CYP1A2, CYP2C19, CYP2C9	Yes
**3g**	375.81	4.42	74.48	Yes	High	CYP1A2, CYP2C19, CYP2C9	Yes
**3h**	342.35	2.90	87.37	No	High	CYP1A2, CYP2C19, CYP2C9	Yes
**3i**	331.32	3.17	87.62	No	High	CYP1A2, CYP2C19, CYP2C9	Yes
**3j**	363.39	2.98	97.27	No	High	CYP1A2, CYP2C19, CYP2C9	Yes
**3k**	350.80	4.64	50.69	Yes	High	CYP1A2, CYP2C19, CYP2C9	Yes
**3l**	385.24	5.17	50.69	Yes	High	CYP1A2, CYP2C19, CYP2C9	Yes
**3m**	351.79	3.65	63.58	Yes	High	CYP1A2, CYP2C19, CYP2C9, CYP2D6, CYP3A4	Yes
**3n**	340.76	3.83	63.83	Yes	High	CYP1A2, CYP2C19, CYP2C9	Yes
**3o**	372.83	3.71	104.71	No	High	CYP1A2, CYP2C19, CYP2C9	Yes
**4a**	314.34	4.36	48.15	Yes	High	CYP1A2, CYP2C19, CYP2C9, CYP2D6	Yes
**4b**	348.78	4.88	48.15	No	High	CYP1A2, CYP2C19, CYP2C9, CYP2D6,	Yes
**4c**	315.33	3.58	61.04	Yes	High	CYP1A2, CYP2C19, CYP2C9, CYP2D6, CYP3A4	Yes
**4d**	304.30	3.66	61.29	Yes	High	CYP1A2, CYP2C19, CYP2C9, CYP2D6, CYP3A4	Yes
**4f**	339.35	4.04	71.94	No	High	CYP1A2, CYP2C19, CYP2C9, CYP2D6, CYP3A4	Yes
**4g**	373.79	4.62	71.94	No	High	CYP1A2, CYP2C19, CYP2C9, CYP2D6, CYP3A4	Yes
**4h**	340.33	3.39	84.83	No	High	CYP1A2, CYP2C19, CYP2C9, CYP2D6, CYP3A4	Yes
**4i**	329.31	3.45	85.08	No	High	CYP1A2, CYP2C19, CYP2C9, CYP2D6, CYP3A4	Yes
**4k**	348.78	4.73	48.15	No	High	CYP1A2, CYP2C19, CYP2C9, CYP2D6,	Yes
**4l**	383.23	5.31	48.15	No	High	CYP1A2, CYP2C19, CYP2C9	Yes
**4m**	349.77	4.01	61.04	Yes	High	CYP1A2, CYP2C19, CYP2C9, CYP2D6, CYP3A4	Yes
**4n**	338.74	4.16	61.29	No	High	CYP1A2, CYP2C19, CYP2C9, CYP2D6, CYP3A4	Yes

^a^ Molecular weight (recommended value <500). ^b^ The logarithm of the compound’s partition coefficient between n-octanol and water (recommended value <5). ^c^ Polar surface area (recommended value ≤140Å2). ^d^ Indicates whether the molecule crosses the blood-brain barrier. ^e^ Degree of gastrointestinal absorption. ^f^ Represents CYP450 subtype inhibition. ^g^ Indicates whether or not the compound fulfills Lipinski’s Rule of Five.

## Data Availability

Data are contained within the article; additional data are available from the corresponding author upon request.

## Sample Availability

Samples of the compounds are not available from the authors.
